# Routine Measurement of Thyroid Stimulating Hormone in Patients Presenting With Third-Degree Atrioventricular Block: Do We Really Need It?

**DOI:** 10.7759/cureus.12712

**Published:** 2021-01-14

**Authors:** Muhammad Faisal, Zubair Mumtaz, Abdul Mueed, Sajid Ali, Haseeb H Raza, Samra Khan, Sayeda Salma, Mustajab Mujtaba, Musa Karim, Faisal Qadir

**Affiliations:** 1 Electrophysiology, National Institute of Cardiovascular Diseases, Karachi, PAK; 2 Medicine, Liaquat National Hospital, Karachi, PAK; 3 Cardiology, National Institute of Cardiovascular Diseases, Karachi, PAK; 4 Research, National Institute of Cardiovascular Diseases, Karachi, PAK

**Keywords:** thyroid stimulating hormone, tsh, atrioventricular block, complete heart block, permanent pacemakers

## Abstract

Background

Hypothyroidism can be a cause of sinus bradycardia. However, thyroid laboratory evaluation is often performed routinely in patients with complete heart block (CHB) though there is little data to support this practice. This study aimed to assess the frequency of thyroid dysfunction in patients presenting with CHB without clinical features of hypothyroidism.

Methodology

All patients referred for permanent pacemaker implantation for CHB were included in this cross-sectional study. Patients with known thyroid disorder or clinical features of thyroid disorder were excluded. Demographic, electrocardiography (EKG), and routine thyroid stimulating hormone (TSH) screening results were recorded.

Results

A total of 102 patients with complete atrioventricular (AV) block were enrolled in the study of which 50.0% (51) were male. The mean age was 61.09 ± 11.74. Co-morbidities included diabetes mellitus 44.1% (45), smoking 36.3% (37), and hypertension 55.9% (57). Mean EKG atrial rate was 82.97 ± 31.31 mmHg with a mean ventricular escape rate of 36.17 ± 5.93. Permanent pacemakers were implanted in all of the patients. Only one patient had an abnormal TSH.

Conclusions

We found a very low prevalence of thyroid dysfunction among patients without clinical features of thyroid dysfunction presenting with third-degree AV block. This calls for cautious prescription of thyroid testing in clinically euthyroid patients.

## Introduction

Although sinus bradycardia is a well-known manifestation of hypothyroidism, there is a paucity of data to suggest a direct relationship of atrioventricular (AV) block with hypothyroidism. Thyroid hormone affects the cardiovascular system through direct or indirect mechanisms. Thyroid hormones have inotropic and chronotropic effects similar to adrenaline, leading to increased heart rate and cardiac output [1]. The overall prevalence of hypothyroidism ranges from 1% to 2%, rising to 7% in individuals aged between 85 and 89 years [2].

Worldwide, the prevalence of third-degree AV block is 0.04% [3]. Limited case reports of patients with known thyroid abnormality and presenting with AV block have been published. However, there are no data to suggest that there is a benefit of routinely assessing thyroid status in clinically euthyroid patients with complete heart block (CHB).

Current guidelines for bradycardia suggest laboratory tests (e.g. thyroid function tests, Lyme titer, potassium, pH) for a potential underlying cause as reasonable only if there is clinical suspicion [3]. However many patients with CHB have routine screening by thyroid stimulating hormone (TSH). This can lead to unnecessary use of resources and is unlikely to cause a change in management plan even if abnormal. This study was aimed to assess the frequency of abnormal TSH levels in patients presenting with CHB without overt clinical thyroid dysfunction.

## Materials and methods

A cross-sectional study conducted from September 2019 to February 2020 at the Department of Adult Cardiology, National Institute of Cardiovascular Diseases (NICVD), Karachi, Pakistan. Adult patients without clinical features of thyroid dysfunction with third-degree AV block, referred for permanent pacemaker implantation, were included in the study. The patients with heart block associated with acute myocardial infarction, drugs, or other reversible causes as well as patients with known thyroid disease or clinical evidence of hypothyroidism or hyperthyroidism were excluded. Patients’ clinical suspicion for hypothyroidism or hyperthyroidism was based on the history of heat/cold intolerance, weight gain/loss, and hair loss. Demographic and routine clinical and laboratory data were recorded, which included TSH assessment. Patients were categorized as having normal or abnormal TSH based on normal reference range of 0.4-4 mI U/L.

The study was approved by the ethical review committee of the NICVD, Karachi, Pakistan. Verbal consent was obtained from all the patients regarding their participation in the study. After ruling out the reversible causes, all the patients with CHB were implanted with permanent pacemakers (PPM) (single or dual chamber) as per the institutional protocol and guidelines. Patients with abnormal TSH were to be referred to an endocrinologist for further assessment and management.

All the data were collected on a predesigned structured proforma and statistical package for social sciences (SPSS 21) was used for the analysis of data. Descriptive statistics, such as mean ± standard deviation (SD), were calculated for quantitative (continuous) variables and frequency and percentages were calculated for categorical variables.

## Results

A total of 102 patients who presented with complete AV block were enrolled in the study; 50.0% (51) were female. The mean age was 61.09 ± 11.74 years. Co-morbidities included diabetes mellitus 44.1% (45), smoking 36.3% (37), and hypertension 55.9% (57) (Table 1).

**Table 1:** Baseline characteristics of patients

Characteristics	Total (n = 102)
Gender	
Male	50% (51)
Female	50% (51)
Age (years)	
Mean ± standard deviation	61.09 ± 11.74 years
Up to 60 years	51% (52)
>60 years	49% (50)
Clinical history	
Diabetes	44.1% (45)
Smoking	36.3% (37)
Hypertension	55.9% (57)

Mean EKG atrial rate was 82.97 ± 31.31 with a mean ventricular escape rate of 36.17 ± 5.93. All the patients were implanted with PPM. Abnormal TSH was noted in one patient (1%) (Table 2). Patient was a 60-year-old male with diabetes and hypertension and normal ejection fraction. His EKG showed CHB with sinus tachycardia at 125 bpm with left bundle branch block (LBBB) type ventricular escape at a rate of 30 bpm.

**Table 2:** Clinical characteristics of patients

Characteristics	Total (n = 102)
Atrial rate	82.97 ± 31.31
Ventricular rate	36.17 ± 5.93
Ejection fraction (%)	53.61 ± 9.21
Type of pacemaker	
Single chamber pacemaker	42.2% (43)
Dual chamber pacemaker	57.8% (59)
Thyroid stimulating hormone (TSH)	
Normal	99% (101)
Elevated	1% (1)

## Discussion

This study, to best of our knowledge, is first of its kind to assess the prevalence of thyroid dysfunction among clinically euthyroid patients with third-degree block. We found that thyroid dysfunction (hypothyroid) was present only in one patient out of 102 studied.

A number of conditions have been reported to be associated with AV block. These include, but not limited to, raised systolic blood pressure and fasting glucose levels [4], PR interval prolongation [5], right [6] and left [7] bundle branch block (BBB), have been linked to severe forms of AV block demanding a pacemaker. Lenegre [8] or Lev [9] disease is an idiopathic and progressive degeneration of cardiac conduction system and estimated to account for about half of AV blocks. Several conditions can lead to conduction system fibrosis and sclerosis, and these conditions are often difficult to be distinguished clinically [10]. Normal aging process to some extent also leads to fibrosis and sclerosis. The prevalence increases with age and there is male predominance with 2:1 male:female ratio [11]. Bradycardia in patients with sinus node dysfunction (SND) and AV block can be due to different etiologies such as hypothyroidism, rheumatologic disorders, and infectious disorders.

Thyroid hormone has effects on heart and blood vessels through various mechanisms. However, the role of thyroid dysfunction in cardiac conduction abnormalities is still not well studied. Case reports have shown improvement in cardiac conduction after treatment of clinically manifested thyroid dysfunctions [12, 13]. Other case reports have also shown that even subclinical hypothyroidism can have negative effects on cardiac conduction, and treatment with Levothyroxine can reverse or improve the condition [14, 15]. A recent study, however, showed that only a quarter of AV block patients with hypothyroidism showed an improvement after levothyroxine treatment [16]. Another study showed that there was no effect of levothyroxine on AV conduction abnormalities [17]. This raises a question on the reversibility of hypothyroidism-related AV block with treatment. 

In our population, Zeb et al. reported that there was no evidence of thyroid dysfunction in patients presenting with high-grade AV block [18]. Another study by Rasheed et al. reported hypothyroidism in a fraction of patients (3.0%) with AV block [19] but did not state how many of these were already diagnosed or had low thyroid levels at the time of the AV block. Our study looked at only new cases of hypothyroidism in patients with CHB and this was only 1%.

This is an important finding and has clinical practice implications especially in a resource-limited environment. In addition to the financial cost, pending test results can increase the hospital stay. Moreover, there is no data to suggest that making a patient euthyroid will obviate the need for pacing making this test redundant.

Although clinical manifestation of hyperthyroidism are more on cardiovascular system, but its effect on AV conduction system is rare and is generally found to be associated with other conditions [16]. In our sample, none of the patients had hyperthyroidism. This could be due to the fact that subclinical hyperthyroidism is less prevalent in the population [20,21]. A previous study has reported that only three out of 21 patients of hyperthyroidism associated AV block responded to antithyroid medication [16]. Furthermore, among these who responded, two patients had pacemaker implantation because of non-response in the initial phase of treatment. This further indicates a very low utility of thyroid function testing in the management of AV block patients because of low yield and unchanged course of management.

In this study, we explored the prevalence of thyroid dysfunction among third-degree AV block patients from a large cardiac center. There are certain methodological limitations that need to be considered while interpreting the results of this study. First, this was a single center study and the sample size was small which may limit the generalizability of the study.

## Conclusions

We found a very low prevalence of thyroid dysfunction among patients without clinical features of thyroid dysfunction presenting with third-degree AV block. This calls for cautious prescription of thyroid testing in clinically euthyroid patients. Further large-scale studies are required to validate the findings of this study and generate high-quality evidence to guide practice.
